# Foreign Body Granuloma After Polylactic Acid Filler Injection for Penile Girth Enhancement: Three Cases Report

**DOI:** 10.1002/ccr3.70811

**Published:** 2025-08-19

**Authors:** Mykola Boiko, Oleksandr Boiko, Serhiy Pasyechnikov, Mykola Notsek

**Affiliations:** ^1^ Clinic “Androcentr” Kyiv Ukraine; ^2^ University Hospital of Cruces Barakaldo Spain; ^3^ O.O. Bogomolets National Medical University Kyiv Ukraine; ^4^ Kyiv International University Kyiv Ukraine

**Keywords:** dermal fillers, foreign body granuloma, hyaluronic acid, injections, penis, polylactic acid

## Abstract

Penile girth enhancement is achieved using a variety of fillers, including autologous fat injection, silicone, collagen, hyaluronic acid, polylactic acid (PLA), polymethylmethacrylate, and dextran. While PLA has a good filling effect due to its bio‐stimulatory properties, it may cause complications in a certain percentage of cases. We describe three patients who developed granulomatous reactions at least 2 months after receiving subcutaneous injections of PLA‐based fillers. In two of the three cases, surgical removal of the infiltrate was necessary due to insufficient response to conservative treatment. Histological examination revealed unspecific chronic granulomatous inflammation of soft tissues. Based on these cases, we conclude that the causes of granuloma formation and ways to prevent it still require further research. Aesthetic specialists should carefully select patients, materials, and application techniques. Patients should be informed about the possible complications of penile girth enhancement.

**Summary**

- One of the most severe complications after polylactic acid filler injection is granuloma formation.
- Aesthetic specialists must be very attentive in selecting the patient, material, and injection technique while performing penile girth enhancement.
- Patients should also be well‐informed about the possible complications of this procedure.

## 1 Introduction

Both surgical and minimally invasive methods are used to address medical or psychological concerns associated with “small penis syndrome” [1]. Filler injection remains among the safest approaches. Various fillers have been used for penile girth enhancement, including autologous fat, silicone, collagen, hyaluronic acid (HA), polylactic acid (PLA), polymethylmethacrylate, and dextran etc. [2].

Facial rejuvenation and, more recently, penile augmentation have shown satisfactory volumizing effects with the use of PLA [3, 4], but are also associated with some complication rates in both facial and penile applications [4, 5]. Unlike other fillers such as HA, PLA stimulates fibroblast proliferation and collagen production, resulting in extended results [6]. The combination of PLA with HA can provide an immediate effect, although delayed adverse events may occur [7].

This report presents three cases of granulomatous reactions that developed at least 2 months post‐injection with PLA‐based fillers.

## 2 Methods

The procedure was conducted in an outpatient setting under local anesthesia, with 2% lidocaine administered at the penile base. Non‐cross‐linked HA was added to the PLA‐based filler to mitigate the temporary absence of filling effect. Injections were administered at the 2 and 10 o'clock positions, lateral to the neurovascular bundle in the distal penile shaft, to minimize the risk of injury. A 20 mL injection (315 mg PLA diluted in 20 mg of non‐cross‐linked HA) was administered between the Dartos and Buck's fascia using a cannula. The penile shaft was gently massaged to promote even filler distribution. Patients were advised to wear an elastic bandage for 3–7 days and abstain from sexual activity and masturbation for 2–3 weeks to facilitate proper healing. The interventions were performed by the same dedicated team with specific training and experience in the procedure.

## 3 Case #1

### 3.1 Case History

A 71‐year‐old patient presented with a dorsal‐lateral subcutaneous induration in the mid‐shaft of the penis, noted 2 months post‐injection. Although asymptomatic during intercourse, the nodule caused psychological discomfort.

### 3.2 Investigations and Treatment

**FIGURE 1:**
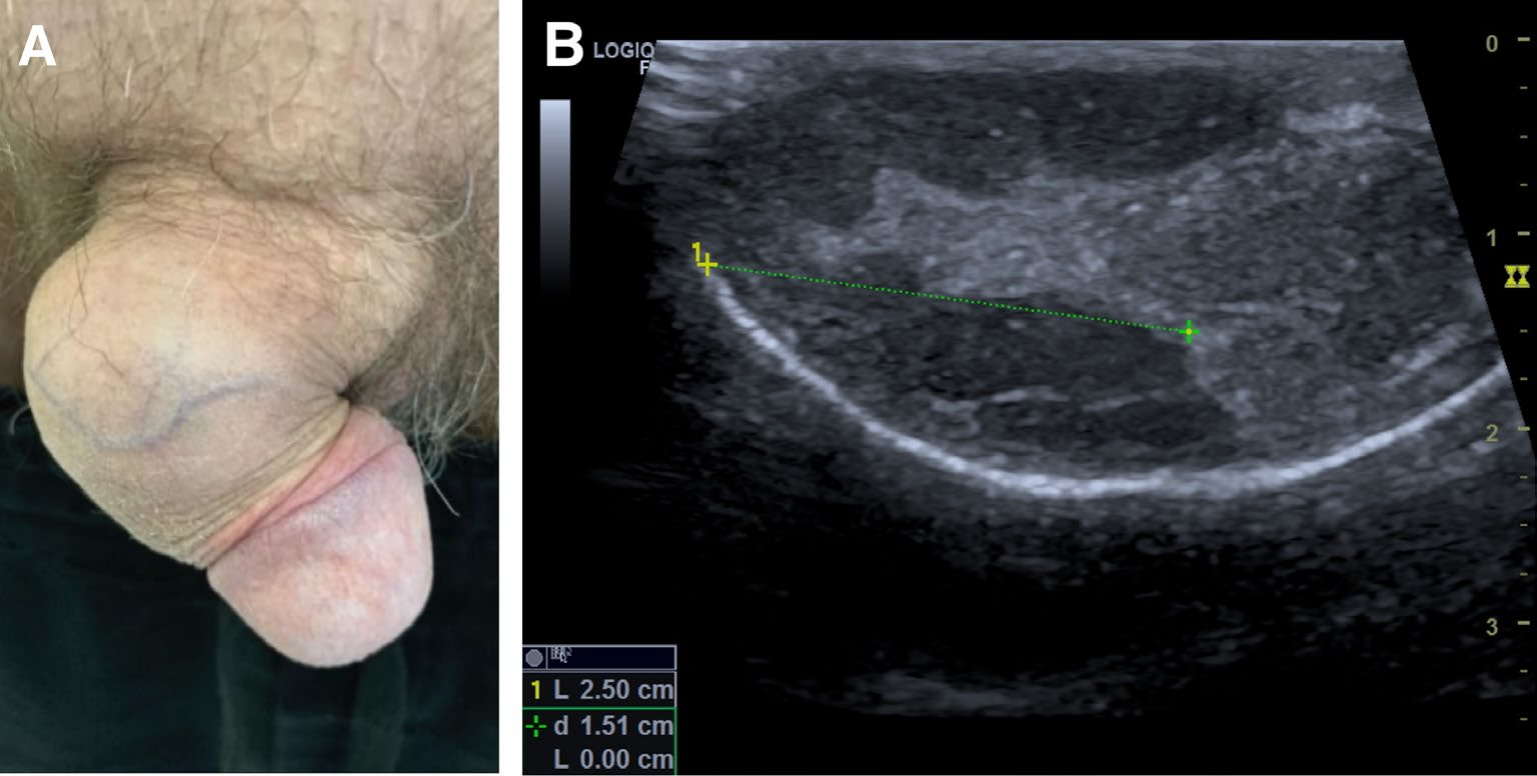
First case. (A) dorsal‐lateral subcutaneous penile granuloma in the middle third of the penis, (B) ultrasound image.

The lesion measured 4 × 2 cm, had a firm consistency, was painless, and was not adherent to the cavernous body (Figure 1A). Ultrasound examination identified a fibrous nodule, measuring 45 × 17 × 22 mm, situated under the skin between superficial and deep fascia in the distal to mid‐penile regions. The nodule exhibited decreased, heterogeneous echogenicity with well‐defined borders (Figure 1B).

Treatment included intralesional hyaluronidase and collagenase injections, along with three betamethasone injections, reducing the infiltrate size from 28.2 to 16.8 cm^3^. The patient has failed conservative therapy.

### 3.3 Results

Surgical excision was required due to an inadequate response to conservative therapy.

Histological analysis revealed focal granulomatous inflammation characterized by numerous histiocytes and multinucleated giant cells, indicative of chronic granulomatous inflammation within soft tissues. Dressings were changed every 2 days post‐surgery for 2 weeks, resulting in successful healing.

## 4 Case #2

### 4.1 Case History

A 34‐year‐old male presented with a nodule on the right lateral aspect of the middle part of his penis 3 months after theprocedure.

### 4.2 Investigations and Treatment

**FIGURE 2:**
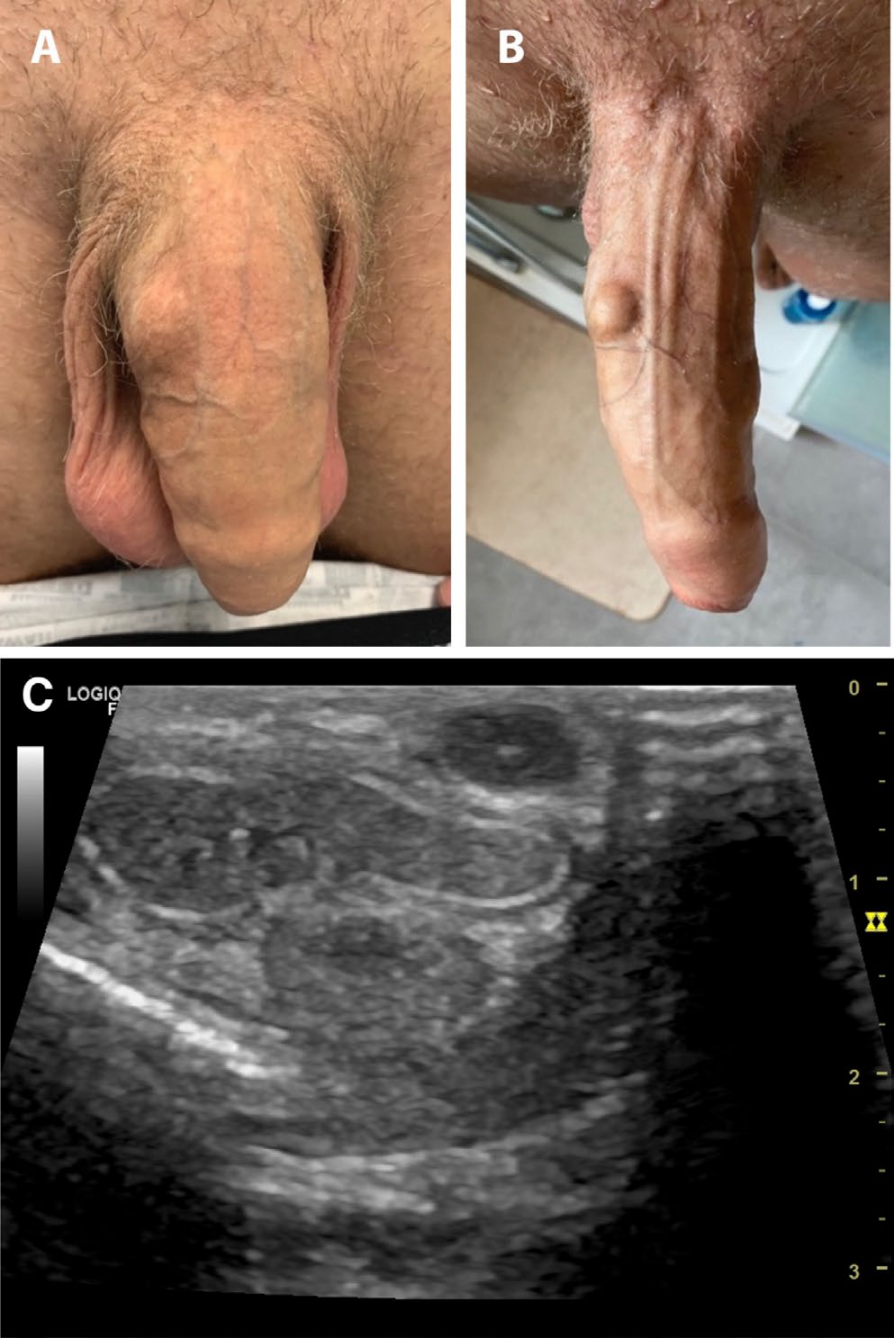
Second case. Nodule on the right lateral part of his penis in: (A) flaccid state, (B) erect state, (C) ultrasound image.

The nodule was painless, had a hard consistency, and was not attached to the skin and cavernous body and situated between superficial and deep fascia. It measured 1 × 2 × 1.5 cm in size and was not visible in the flaccid state but caused discomfort during sexual intercourse (Figure 2A–C).

The patient underwent a course of intranodal hyaluronidase and corticosteroid injections, which yielded minimal improvement; the patient has failed conservative therapy.

### 4.3 Results

Surgical excision of the nodule was subsequently performed, with microscopic analysis revealing focal chronic granulomatous inflammation. Dressings were changed every 2 days post‐surgery for 2 weeks, resulting in successful healing. Six months post‐surgery, the patient received corrective penile augmentation using HA filler injections.

## 5 Case #3

### 5.1 Case History

A 24‐year‐old patient presented with diffuse subcutaneous induration of his penis 2 months after undergoing PLA injections.

### 5.2 Investigations and Treatment

**FIGURE 3:**
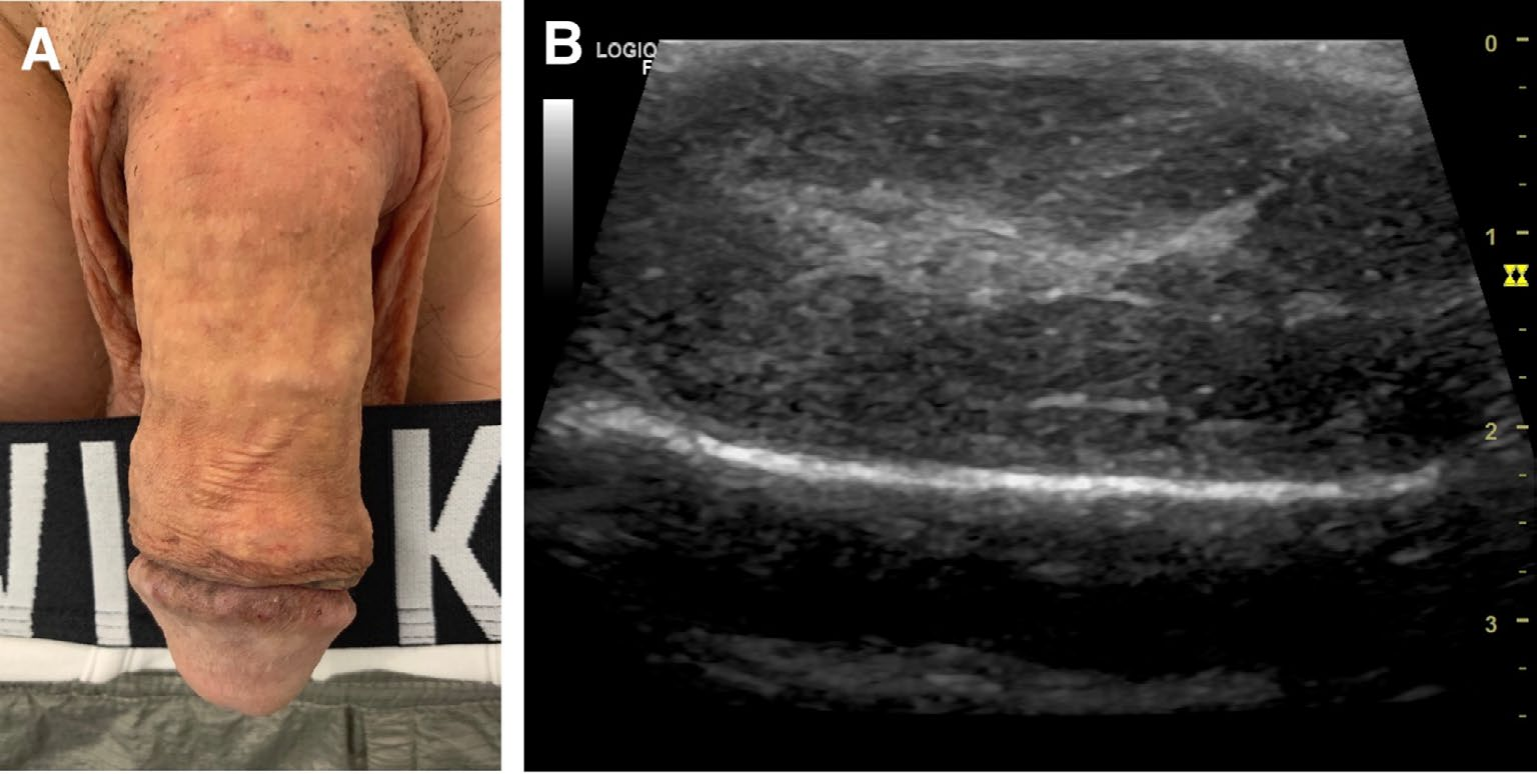
Third case. (A) diffuse subcutaneous induration of the penis, (B) ultrasound image.

Painless bumps were unevenly distributed over the surface of the penis, leading to slight asymmetry in the flaccid state (Figure 3A,B). The patient reported no discomfort during sexual intercourse and was offered asymmetry correction with HA filler.

### 5.3 Results

Standardized follow‐up, identical to that which was used after the initial procedure, was recommended after correction. Three months post‐correction, the patient reported satisfaction with both cosmetic and sexual outcomes.

## 6 Discussion

Penile girth enhancement with filler injections has shown good results and high satisfaction rates. However, it comes with a certain rate of complications [4, 5]. The complications after filler injection include three main types: those related to the patient, to the procedural technique, and filler characteristics [8].

The ideal material for soft tissue augmentation should be biocompatible, non‐antigenic, non‐pyrogenic, non‐inflammatory, non‐toxic, easy to administer, stable, and cost‐effective [9]. Substances like mineral oils, vaseline, metallic mercury, and paraffin are associated with high complication rates, potentially resulting in penile shortening, deformities, infections, necrosis, and erectile dysfunction [10, 11, 12]. Safer alternatives include HA, polymethyl methacrylate microspheres, calcium hydroxylapatite, and PLA fillers [13, 14]. In a review by Daines et al. analyzing 2089 injections with HA, PLA, and calcium hydroxylapatite, 14 complications were identified, half of which were granulomas, with PLA showing the highest incidence [15]. Conversely, a study on host defense reactions to 10 commonly used materials in aesthetic medicine demonstrated excellent biocompatibility for PLA, alongside collagen, HA, polymethylmethacrylate, dimethylpolysiloxane, dextran microspheres, hydroxyethylmethacrylate, polyacrylamide, polyvinylhydroxide, and calcium hydroxylapatite [16].

The interval between foreign body implantation and granuloma formation varies widely and may extend to several years in certain cases [6, 17]. Abnormal recipient reactivity has been documented, particularly in patients with comorbidities such as sarcoidosis [15].

Minimizing complication risk requires selecting safe, well‐studied fillers with low complication rates. Injections should be avoided in patients with granulomatous diseases, immunocompromised status, or prior granuloma history. Technique‐related risks include superficial injection, excessive volume, and accidental intravascular injection [9]. Specific recommendations can reduce granuloma incidence: use an 18–22 gauge needle, apply filler uniformly in multiple directions without repeating channels, limit initial filler volume to 20 mL, and gently massage the area for at least 5 days post‐injection. An elastic bandage should be worn for 5 days to minimize edema, and patients are advised to avoid sexual activity and masturbation for 2–3 weeks.

Anatomopathological analysis revealed nonspecific granulomatous inflammation. All patients had no relevant medical history; procedures adhered strictly to protocol, leaving the cause of granuloma formation undetermined.

The therapeutic options for treating complications include the use of imiquimod gel, hyaluronidase injection, collagenases, and corticosteroids with or without 5‐fluorouracil [18, 19]. Surgical removal is recommended in cases where these treatments are ineffective [1].

Further research is needed to elucidate the mechanisms underlying granuloma formation and strategies for its prevention. Aesthetic specialists should exercise caution in patient selection, material choice, and injection technique. Patients must also be thoroughly informed about potential complications associated with penile girth enhancement.

## Author Contributions

**Mykola Boiko:** conceptualization, data curation, methodology, supervision. **Oleksandr Boiko:** data curation, formal analysis, validation, visualization. **Serhiy Pasyechnikov:** resources, supervision, validation, writing – review and editing. **Mykola Notsek:** project administration, supervision, visualization, writing – review and editing.

## Consent

Written informed consent was obtained from all three patients to publish this report in accordance with the journal's patient consent policy.

## Conflicts of Interest

The authors declare no conflicts of interest.

## Data Availability

The data that support the findings of this study are available from the corresponding author upon reasonable request.
